# Effective timing of hyaluronate gel injection in image-guided adaptive brachytherapy for uterine cervical cancer: a proposal of the ‘adjusted dose score’

**DOI:** 10.1093/jrr/rrae031

**Published:** 2024-05-13

**Authors:** Yusaku Miyata, Etsuyo Ogo, Kenta Murotani, Naotake Tsuda, Gen Suzuki, Chiyoko Tsuji, Ryosuke Akeda, Koichiro Muraki, Chikayuki Hattori, Toshi Abe

**Affiliations:** Department of Radiology, Kurume University School of Medicine, 67 Asahimachi, Kurume, Fukuoka 830-0011, Japan; Department of Radiology, Kurume University School of Medicine, 67 Asahimachi, Kurume, Fukuoka 830-0011, Japan; Biostatistics Center, Kurume University, 67 Asahimachi, Kurume, Fukuoka 830-0011, Japan; Department of Obstetrics and Gynecology, Kurume University School of Medicine, 67 Asahimachi, Kurume, Fukuoka 830-0011, Japan; Department of Radiology, Graduate School of Medical Science, Kyoto Prefectural University of Medicine, 465 Kajiicho Kawaramachi Hirokoji, Kamigyo-ku, Kyoto 602-8566, Japan; Department of Radiology, Kurume University School of Medicine, 67 Asahimachi, Kurume, Fukuoka 830-0011, Japan; Department of Radiology, Kurume University School of Medicine, 67 Asahimachi, Kurume, Fukuoka 830-0011, Japan; Department of Radiology, Kurume University School of Medicine, 67 Asahimachi, Kurume, Fukuoka 830-0011, Japan; Department of Radiology, Kurume University School of Medicine, 67 Asahimachi, Kurume, Fukuoka 830-0011, Japan; Department of Radiology, Kurume University School of Medicine, 67 Asahimachi, Kurume, Fukuoka 830-0011, Japan

**Keywords:** adjusted dose score, cervical cancer, hyaluronate gel injection, image-guided adaptive brachytherapy, rectovaginal septum, vesicovaginal septum

## Abstract

Hyaluronate gel injection (HGI) in the rectovaginal septum and vesicovaginal septum is effective in the setting of high-dose-rate image-guided adaptive brachytherapy (IGABT) for cervical cancer. We aimed to retrospectively investigate optimal conditions for HGI to achieve optimal dose distribution with a minimum number of HGI. We classified 50 IGABT plans of 13 patients with cervical cancer who received IGABT both with and without HGI in the rectovaginal septum and vesicovaginal septum into the following two groups: plan with (number of plans = 32) and plan without (number of plans = 18) HGI. The irradiation dose parameters of high-risk clinical target volume (CTV_HR_) and organs at risk per fraction were compared between these groups. We also developed the adjusted dose score (ADS), reflecting the overall irradiation dose status for four organs at risk and CTV_HR_ in one IGABT plan and investigated its utility in determining the application of HGI. HGI reduced the maximum dose to the most exposed 2.0 cm^3^ (D_2.0 cm3_) of the bladder while increasing the minimum dose covering 90% of CTV_HR_ and the percentage of CTV_HR_ receiving 100% of the prescription dose in one IGABT plan without causing any associated complications. An ADS of ≥2.60 was the optimum cut-off value to decide whether to perform HGI. In conclusion, HGI is a useful procedure for improving target dose distribution while reducing D_2.0 cm3_ in the bladder in a single IGABT plan. The ADS can serve as a useful indicator for the implementation of HGI.

## INTRODUCTION

Image-guided adaptive brachytherapy (IGABT) is an essential part of radiotherapy for cervical cancer, as it involves the delivery of high-dose radiation to the tumor. Minimizing the dose to adjacent normal tissue (organs at risk [OARs]) and mitigating the occurrence of late adverse events while maintaining the dose to the intended treatment area (high-risk clinical target volume [CTV_HR_]) are crucial. To achieve this objective, spacers are used in some cases to establish a physical separation between the CTV_HR_ and OARs. Kishi *et al.* and another Japanese group have reported the effectiveness of hyaluronate gel (Suvenyl®; Chugai Pharmaceutical Co., Tokyo, Japan) as a spacer for high-dose-rate IGABT in cervical cancer [1–7].

However, optimal dose distribution can be achieved in radiotherapy for cervical cancer without hyaluronate gel injection (HGI) in some cases. Additionally, the use of HGI is not covered by insurance in Japan, and medical providers are responsible for HGI costs. Therefore, achieving optimal dose distribution with the lowest HGI frequency possible is preferable. Previous studies [1–7] have demonstrated the use of spacers with HGI with Suvenyl® for cervical cancer but have not indicated the optimal timing for HGI administration to minimize its frequency.

Therefore, this retrospective study aimed to determine eligibility criteria to effectively utilize HGI from resource and monetary perspectives. To this end, we compared the adjusted dose score (ADS), which was invented to centrally evaluate the relationship between irradiation dose and dose constraint for the CTV_HR_ and each of the four OARs (the rectum, bladder, sigmoid colon and small intestine), in an IGABT plan before and after HGI implementation in patients with cervical cancer who received HGI with Suvenyl® in the middle of multiple IGABTs. Further, we determined whether the ADS could serve as an indicator of HGI implementation to achieve optimal dose distribution with a minimum number of HGIs.

## MATERIALS AND METHODS

### Patients

Medical records of 53 patients with pathologically diagnosed cervical cancer who underwent radical radiotherapy with IGABT in our institution between April 2022 and August 2023 were retrospectively reviewed. We aimed to determine the optimal conditions for HGI implementation. Therefore, this study included patients with cervical cancer who did not receive HGI at the first IGABT session and who received HGI at the second or subsequent IGABT sessions, as it was anticipated that repeat IGABT without HGI would not meet the specified dose constraints for the CTV_HR_ and OARs, as shown in previous clinical trial protocols [8]. A total of 35 patients who did not receive HGI during IGABT and 5 who received HGI during all brachytherapy sessions were excluded from this study. The remaining 13 patients were included, and 50 IGABT plans from these patients were investigated. Although the optimal scenario involved initiating HGI at the second IGABT session, 5 of the 13 patients received HGI at the third IGABT session. This delay was attributed to the necessity of seeking approval for off-label use of HGI from the University Hospital Ethics Committee, which was not obtained in time for the second IGABT session due to the prolonged process of obtaining patient consent or the extensive application process.

### Radiotherapy details, methods of HGI and follow-up

All patients received external beam radiotherapy (EBRT) and IGABT. Regarding EBRT, whole-pelvic radiotherapy (WPRT) was performed using the box irradiation technique with high-energy 10-MV X-ray photons from a linear accelerator at a daily fraction of 1.8 Gy delivered five times weekly. After administering 30–40 Gy of WPRT, 10–20 Gy of WPRT with a central shield (CS) using a 40-mm-wide block, made using a 5-mm-wide multileaf collimator of a linear accelerator, reduced the rectum and bladder dose until the pelvic sidewall received 50.4 Gy. The upper boundary of the CS was determined to be 5 mm inferior to the lower border of the presacral lymph node chain. Metastases, if any, in the para-aortic lymphatic chains were also included in the irradiation field. All EBRTs were planned using the following treatment planning systems: RayStation® (RaySearch Laboratories AB, Stockholm, Sweden) or Eclipse® (Varian Medical Systems, Inc., Palo Alto, CA, USA).

After CS implementation, IGABT was administered using tandem, ovoid or cylinder applicators, as well as additional interstitial needles inserted transperineally or transvaginally for large or irregularly shaped tumors (intracavitary and interstitial [IC/IS] brachytherapy). All needles were 6-Fr ProGuide® sharp plastic needles (240 or 294 mm in length; Nucletron, an Elekta company, Elekta 83 AB, Stockholm, Sweden). These applicators or needles were inserted with the patients in the lithotomy position and guided by transrectum ultrasonography (TRUS) under transvenous sedation and sacral epidural anesthesia. Computed tomography (CT) scans were conducted using the Aquilion® LB system from Canon, Tokyo, Japan. Patients were positioned in the leg extension posture with applicators securely in place and remained immobile throughout the procedure. IGABT planning utilized these CT images, which were acquired with a slice interval of 2 mm. The delineation of CTV_HR_ and OARs with CT images was based on the Japanese Radiation Oncology Study Group guidelines [9]. The sigmoid colon and small intestine were delineated caudally to the upper margin of the uterus. When differentiating the sigmoid colon from the small intestine surrounding the uterus proved challenging, the distinction was made by comparing set-up CT and magnetic resonance (MR) images acquired 1 week before the initial IGABT or by following the trajectory of the sigmoid colon from the descending colon to the anus. Treatment planning was conducted using a brachytherapy planning system (Oncentra®, Nucletron, Veenendaal, Netherlands). In every IGABT session, the reference dose per fraction prescribed was 6.0 Gy, and every plan was optimized to ensure that the 6.0 Gy isodose covered the CTV_HR_ while adhering to OAR dose restrictions, with reference to the dose targets reported in prospective clinical trials conducted in Japan [8]. The minimum dose covering 90% of the CTV_HR_ (CTV_HR_D_90%_) and percentage of the CTV_HR_ receiving 100% of the prescription dose (6.0 Gy) (CTV_HR_V_100%_) were used as the index for coverage of the target, as well as the maximum dose to the most exposed 2.0 cm^3^ (D_2.0 cm3_) of OARs as index values for the OAR dose evaluation. Dose distribution was adjusted to ensure that CTV_HR_D_90%_ > 7.0 Gy, D_2.0 cm3_ of the rectum <5.5 Gy, and D_2.0 cm3_ of the bladder were <6.5 Gy per fraction. However, the OARs in this study were the rectum, bladder, sigmoid colon and small intestine. In contrast, previous studies have not specified dose constraints for each IGABT session in the sigmoid colon and small intestine. Therefore, in this study, the recommendation of D_2.0 cm3_ of the sigmoid colon and small intestine was set at <5.5 and <5.0 Gy per fraction, respectively. CTV_HR_V_100%_ of 100%, or as close to 100% as possible, was considered the ideal dose coverage. To sum the total doses for EBRT and IGABT and evaluate the total CTV_HR_D_90%_ and total D_2.0 cm3_ of each OAR, the equivalent dose in 2 Gy fractions (EQD2) was calculated using the following formula based on the linear-quadrate model [10–12]:


$$ \mathrm{EQD}2=\mathrm{D}\ \left\{\mathrm{d}+\left(\mathrm{\alpha} /\mathrm{\beta} \right)\right\}/\left\{2+\left(\mathrm{\alpha} /\mathrm{\beta} \right)\right\} $$

where D represents the total dose, d denotes the dose per fraction, α/β = 10 Gy for CTV_HR_ and α/β = 3 Gy for OARs.

However, the irradiation dose of EBRT to the CTV_HR_, rectum and bladder after CS implementation was not accounted for in the calculations in this study. The total CTV_HR_D_90%_ and D_2.0 cm3_ of the rectum and bladder were also based on the dose constraints of the aforementioned clinical trial [8]. The total target values for CTV_HR_D_90%_, D_2.0 cm3_ of the rectum, and D_2.0 cm3_ of the bladder were >70, <65 and <75 Gy, respectively. The total target values for D_2.0 cm3_ of the sigmoid colon and small intestine were decided to be <65 GyEQD2 and <60 GyEQD2, respectively.

**Fig. 1:**
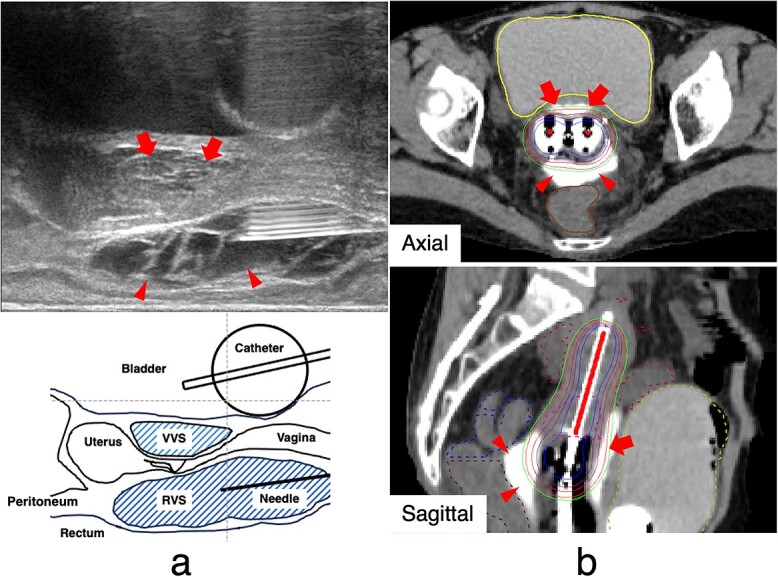
Hyaluronate gel injection. (a) Schema for transrectum ultrasonography-guided hyaluronate gel injection with applicators and hyaluronate gel in the RVS (arrowheads) and VVS (arrows). (b) Axial and sagittal computed tomograms with applicators and hyaluronate gel in the RVS (arrowheads) and VVS (arrows). Hyaluronate gel isolates the rectum and bladder from the high-dose irradiated area of brachytherapy. The magenta, brown, blue, yellow and red dotted lines indicate the high-risk clinical target volume (CTV_HR_), rectum, sigmoid colon, bladder and small intestine, respectively. RVS = rectovaginal septum, VVS = vesicovaginal septum, CTV_HR_ = high-risk clinical target volume.

Suvenyl®, a purified sodium hyaluronate used in HGI, has been demonstrated to be a safe intra-articular injection drug (0.1–2.0% adverse events). It should be injected at each IGABT session as it is absorbed in 2–3 days [6]. HGI is performed during the second and subsequent IGABT sessions at our institution when the dose to the CTV_HR_ is set to the required dose (>7.0 Gy) in the planning of the first IGABT, resulting in a higher dose to the OARs, or conversely, the dose to the CTV_HR_ was <7.0 Gy to comply with the dose constraint for the OARs, and if this plan is repeated, the total dose to the OARs or CTV_HR_ is not expected to meet the dose constraints. However, patients with bladder or rectal involvement are excluded from this indication. After its introduction, HGI was implemented in every IGABT session. The HGI methodology is described as follows. Before inserting the applicators, the 19G needle (a disposable ultrasonography-compatible puncture needle; Create Medic Co., Ltd, Kanagawa, Japan) was advanced to the rectovaginal septum (RVS) and vesicovaginal septum (VVS) through the anterior and posterior vaginal walls, respectively, using TRUS to confirm the position of the needle tip. After confirming the needle position with TRUS, hyaluronate gel was injected into those septa. Hyaluronate gel comprised 12.5 cm^3^ of Suvenyl® (Chugai Pharmaceutical Co., Tokyo, Japan), diluted with 10.5 cm^3^ of saline solution and 2 cm^3^ of contrast media (Iopamidol 370®; Fuji Pharmaceutical Company, Toyama, Japan) and up to a total volume of 25 cm^3^. Of this volume, 10 and 15 cm^3^ were injected into the VVS and RVS, respectively (Fig. 1).

Approximately 1 month after treatment, local tumor response was determined using the Response Evaluation Criteria in Solid Tumors version 1.1, along with the findings of the gynecological examination, pathological examination, CT and MR imaging [13]. Acute adverse events were evaluated according to the National Cancer Institute Common Terminology Criteria for Adverse Events (CTCAE) version 4.03 [14].

### Adjusted dose score

An ideal IGABT plan is adjusted to ensure that a sufficient dose is prescribed for the CTV_HR_ while adhering to the dose constraints for the following four OARs: the rectum, bladder, sigmoid colon and small intestine. Nevertheless, an optimal dose distribution may not always be attainable. Although the aforementioned clinical trial protocols [8] describe these dose constraints independently, the adjusted irradiation dose for each OAR and the CTV_HR_ results from the interaction between each adjustment; that is, each dose is not an independent factor. In IGABT, adjusting the dose indicates prolonging or shortening the dwell time of the radiation source at a given coordinate; increasing the dose for the CTV_HR_ always increases the dose for all OARs, and vice versa. If all dose constraints cannot be met, i.e. if the dose for the CTV_HR_ is decreased under the target to achieve the dose constraint for the OARs, or if the dose constraint for the OARs cannot be met to ensure sufficient dose for the CTV_HR_, evaluating which dose parameter will meet the dose constraint depends on the value judgment of whether the priority is to increase the dose for the CTV_HR_ or to decrease the dose for the OARs, making the wholesale evaluation of the quality of the IGABT plan difficult. Therefore, we invented the following formula, named ‘ADS’, to centrally evaluate the relationship between the irradiation dose and dose constraint for each of the four OARs and CTV_HR_ in a single IGABT plan and to evaluate whether the plan was adequate:

ADS = {Σ [the actual D_2.0 cm3_ per fraction of each OAR (Gy)/the recommended limitation of D_2.0 cm3_ per fraction of each OAR (Gy)]}/[the actual CTV_HR_D_90%_ per fraction (Gy)/the recommended minimum CTV_HR_D_90%_ per fraction (Gy)].

The recommended limitations of D_2.0 cm3_ per fraction of the rectum, bladder, sigmoid colon and small intestine were 5.5, 6.5, 5.5 and 5.0 Gy, respectively. The recommended minimum CTV_HR_D_90%_ per fraction is 7.0 Gy. However, these values are based on clinical trial protocols [8] and should be determined in accordance with the respective indices or guidelines when used in other countries. Illustrations of the calculation are provided below.

(Case 1, favorable example) A case in which the D_2.0 cm3_ per fraction is low for each OAR and the CTV_HR_D_90%_ per fraction is high enough. The actual CTV_HR_D_90%_ per fraction is 9.1 Gy, and the actual D_2.0 cm3_ per fraction of the rectum, bladder, sigmoid colon and small intestine are 3.9, 5.8, 4.2 and 0.8 Gy, respectively,


$$ \mathrm{ADS}=\left[3.9/5.5+5.8/6.5+4.2/5.5+0.8/5.0\right]/\left[9.1/7.0\right]=1.94. $$

(Case 2, unfavorable example) A case in which D_2.0 cm3_ per fraction of some OARs is relatively high, even though the CTV_HR_D_90%_ per fraction is not high enough. The actual CTV_HR_D_90%_ per fraction is 6.8 Gy, and the actual D_2.0 cm3_ per fraction of the rectum, bladder, sigmoid colon and small intestine are 6.1, 5.8, 4.1 and 5.4 Gy, respectively,


$$ \mathrm{ADS}=\left[6.1/5.5+5.8/6.5+4.1/5.5+5.4/5.0\right]/\left[6.8/7.0\right]=3.94. $$

As illustrated above, the formula was designed so that the fewer the dose constraints for each of the OAR and CTV_HR_ are met, the higher the ADS value will be, and vice versa.

### Statistical analyses

A total of 50 plans from 13 patients with cervical cancer were categorized into the following two groups: IGABT plans with (number of plans = 32) and without (number of plans = 18) HGI groups. Considering the application of HGI in this study, an IGABT plan without HGI was a plan for which it was anticipated that the total dose for the OARs or CTV_HR_ would not meet prescribed dose constraints if the plan were repeated.

The CTV_HR_D_90%_, CTV_HR_V_100%_, D_2.0 cm3_ of the OARs (rectum, bladder, sigmoid colon and small intestine), CTV_HR_ and the ADS per fraction were compared between the two groups using the Wilcoxon rank-sum test, and the utility of HGI was assessed in one IGABT plan. However, multiplicity was not assessed due to its exploratory nature. Subsequently, to assess the utility of the ADS in determining the application of HGI, a receiver operating characteristic (ROC) curve analysis was conducted, and the optimal cut-off value on the ROC curve was determined using the Youden Index. Statistical significance was set at *P* < 0.05. All statistical analyses were conducted using RStudio, version 2023.06.2+561 (RStudio: Integrated Development by R. RStudio, Inc., Boston, MA, USA).

## RESULTS

Patients’ characteristics and treatment details are presented in Table 1. The median age of the 13 patients was 61 (range, 42–84) years. Ten, two and one patient had squamous cell carcinoma, adenocarcinoma and adenosquamous cell carcinoma, respectively. Eleven of the thirteen patients received four cycles of IGABT, while two received three cycles. Five of the thirteen patients (19 of the 50 plans) were treated with intracavitary brachytherapy, whereas the remaining eight (31 plans) were treated with IC/IS brachytherapy. All patients received no HGI at the first IGABT; 8 of the 13 patients were introduced to HGI at the second IGABT, whereas 5 were introduced to HGI at the third IGABT, and all patients received two or more cycles of HGI. The total CTV_HR_D_90%_ of all patients was >70 GyEQD2, the recommended target dose.

**Table 1:** Patients’ characteristics and treatment details

**Age (year)**	**FIGO stage (2018)** ^*^	**Histology**	**EBRT** **(WPRT** ^**^ **)**	**IGABT**	**IGABT techniques**	**Number of HGI**	**Total CTV**_**HR**_ **D**_**90%**_ **(GyEQD2)**	**Total D**_**2.0 cm3**_ **of the rectum (GyEQD2)**	**Total D**_**2.0 cm3**_ **of the bladder (GyEQD2)**	**Total D**_**2.0 cm3**_ **of the sigmoid colon (GyEQD2)**	**Total D**_**2.0 cm3**_ **of the small intestine (GyEQD2)**	**Local tumor response**
77	IB1	Adeno	50.4 Gy/28 fr. (39.6 Gy/22 fr.)	6 Gy × 4 fr.	ICBT	2 fr.	85.0	55.7	76.9	64.7	48.2	CR
72	IIIB	SCC	50.4 Gy/28 fr. (30.0 Gy/15 fr.)	6 Gy × 4 fr.	IC/IS	3 fr.	80.4	50.3	62.2	61.9	41.5	CR
42	IIB	SCC	50.4 Gy/28 fr. (30.6 Gy/17 fr.)	6 Gy × 4 fr.	IC/IS	3 fr.	82.9	39.2	57.5	55.2	53.0	CR
84	IIIA	SCC	50.4 Gy/28 fr. (39.6 Gy/22 fr.)	6 Gy × 4 fr.	ICBT	3 fr.	87.1	55.3	69.1	59.7	52.0	CR
49	IIIC1r	SCC	50.4 Gy/28 fr. (39.6 Gy/22 fr.)	6 Gy × 4 fr.	IC/IS	3 fr.	83.4	53.3	60.1	66.0	63.0	CR
50	IIIC2r	SCC	50.4 Gy/28 fr. (45.0 Gy/25 fr.)	6 Gy × 3 fr.	IC/IS	2 fr.	79.9	71.9	64.1	64.3	53.9	PD
73	IIIC1r	SCC	50.4 Gy/28 fr. (30.6 Gy/17 fr.)	6 Gy × 4 fr.	IC/IS	3 fr.	75.7	65.6	69.6	54.2	51.1	PR
50	IIB	Adeno	50.4 Gy/28 fr. (30.6 Gy/17 fr.)	6 Gy × 4 fr.	IC/IS	2 fr.	81.6	43.2	79.6	57.7	32.9	CR
61	IIB	SCC	50.4 Gy/28 fr. (25.2 Gy/14 fr.)	6 Gy × 4 fr.	ICBT	2 fr.	77.6	30.8	62.7	57.3	45.4	CR
62	IIB	SCC	50.4 Gy/28 fr. (45.0 Gy/25 fr.)	6 Gy × 3 fr.	ICBT	2 fr.	82.7	61.4	75.8	69.8	45.3	CR
55	IIIB	SCC	50.4 Gy/28 fr. (30.6 Gy/17 fr.)	6 Gy × 4 fr.	IC/IS	2 fr.	82.0	63.5	75.5	57.1	42.0	CR
69	IIIB	AdSq	50.4 Gy/28 fr. (36.0 Gy/20 fr.)	6 Gy × 4 fr.	IC/IS	2 fr.	95.0	62.9	77.4	62.4	53.0	CR
60	IIB	SCC	50.4 Gy/28 fr. (32.4 Gy/18 fr.)	6 Gy × 4 fr.	ICBT	3 fr.	83.3	61.8	66.6	64.0	38.9	CR

FIGO, International Federation of Gynecology and Obstetrics; Adeno, adenocarcinoma; SCC, squamous cell carcinoma; AdSq, adenosquamous cell carcinoma; EBRT, external beam radiotherapy; WPRT, whole-pelvic radiotherapy; fr., fractions; IGABT, image-guided adaptive brachytherapy; ICBT, intracavitary brachytherapy; IC/IS brachytherapy, intracavitary or interstitial brachytherapy; HGI, hyaluronate gel injection; CTV_HR_, high-risk clinical target volume; EQD2, the equivalent dose in 2 Gy fractions; CTV_HR_D_90%_, the minimum dose covering 90% of the CTV_HR_; D_2.0 cm3_, the most exposed 2.0 cm^3^; CDDP, cisplatin, CR, complete response; PR, partial response; PD, progressive disease.

^*^FIGO stage is based on the 2018 edition for cervical cancer.

^**^The total dose of EBRT is shown in the upper row and the dose of EBRT before insertion of the central shield (WPRT) is shown in the lower row.

The total D_2.0 cm3_ of each OAR in all patients was under the recommended D_2.0 cm3_ as follows: <75 GyEQD2 (rectum), <85 GyEQD2 (bladder), <75 GyEQD2 (sigmoid colon) and <70 GyEQD2 (small intestine). Eleven patients achieved a complete response to radiotherapy, and one had a partial response; however, one patient had a progressive disease. The median follow-up period was 198 (range, 84–444) days, and no acute adverse events in grade 3 or more related to radiotherapy and no complications related to HGI were found.

Among the 50 IGABT plans for the 13 patients, 18 were IGABT plans without HGI (plans that did not meet the total dose constraint) and 32 were IGABT plans with HGI. Table 2 presents the dosimetric parameters, ADS and CTV_HR_ for a single IGABT session between the groups with and without HGI. CTV_HR_D_90%_, CTV_HR_V_100%_ and D_2.0 cm3_ of the bladder significantly differed between the two groups, whereas D_2.0 cm3_ of other OARs and the CTV_HR_ did not differ significantly.

**Table 2:** Comparison of the dosimetric parameters, ADS and CTV_HR_ for a single IGABT session between IGABT plans with and without HGI

**Parameters**	**IGABT plans without HGI (number of plans = 18)**	**IGABT plans with HGI (number of plans = 32)**	***P*-value**
CTV_HR_D_90%_ (GyEQD2)			
Median (IQR)	7.88 (7.43–8.41)	8.33 (7.99–8.85)	0.049
CTV_HR_V_100%_ (%)			
Median (IQR)	100.0 (99.9–100.0)	100.0 (100.0–100.0)	0.042
D_2.0 cm3_ of the rectum (GyEQD2)			
Median (IQR)	4.27 (3.06–4.93)	3.68 (2.62–4.92)	0.363
D_2.0 cm3_ of the bladder (GyEQD2)			
Median (IQR)	5.78 (5.58–6.27)	5.23 (4.31–6.00)	0.037
D_2.0 cm3_ of the sigmoid colon (GyEQD2)			
Median (IQR)	4.52 (4.12–5.00)	4.60 (4.24–4.94)	0.746
D_2.0 cm3_ of the small intestine (GyEQD2)			
Median (IQR)	3.02 (2.41–4.30)	2.59 (1.85–3.71)	0.169
ADS			
Median (IQR)	2.70 (2.46–2.99)	2.37 (2.17–2.52)	0.001
CTV_HR_ (cm^3^)			
Median (IQR)	29.77 (16.86–51.47)	18.88 (16.48–43.41)	0.461

ADS, adjusted dose score; CTV_HR_, high-risk clinical target volume; IGABT, image-guided adaptive brachytherapy; HGI, hyaluronate gel injection; CTV_HR_D_90%_, the minimum dose covering 90% of the CTV_HR_; EQD2, the equivalent dose in 2 Gy fractions; CTV_HR_V_100%_, the percentage of CTV_HR_ receiving 100% of the prescription dose; D_2.0 cm3_, the most exposed 2.0 cm^3^; IQR, interquartile range.

**Fig. 2:**
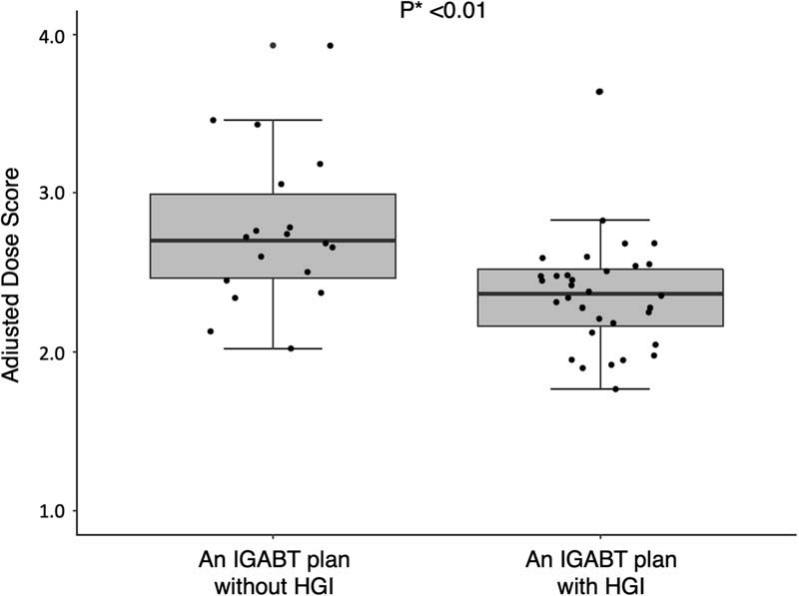
The box-whisker and jitter plots for the distribution of the ADS between the IGABT plans with and without HGI groups. ADS = adjusted dose sore, IGABT = image-guided adaptive brachytherapy, HGI = hyaluronate gel injection. ^*^The ADS of the two groups were compared using the Wilcoxon rank-sum test.

**Fig. 3:**
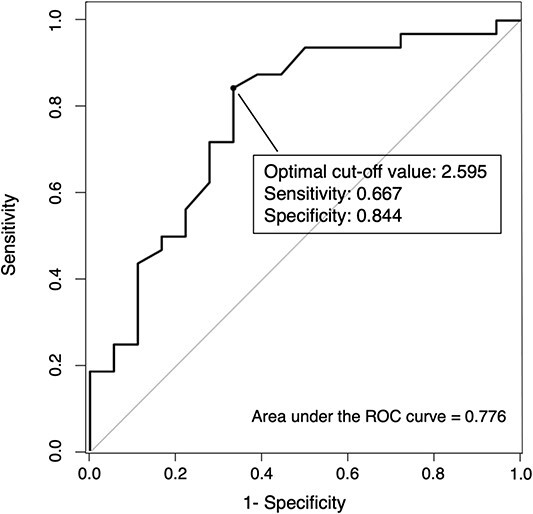
The receiver operating characteristic curve for the ADS, which was determined to be a predictor of optimal conditions for implementing HGI. ADS = adjusted dose sore, HGI = hyaluronate gel injection.

The ADS was significantly lower in the group with HGI than in that without HGI (2.37 vs. 2.70, *P* < 0.01). Figure 2 describes the box-whisker and jitter plots for the distribution of the ADS between the IGABT plans with and without HGI groups. Based on the ROC analysis (Fig. 3), an ADS of ≥2.60 was determined to be the optimum cut-off value to decide whether to perform HGI, with an area under the curve of 0.78 (95% confidence interval [CI]: 0.63–0.92, sensitivity = 66.7%, specificity = 84.4%).

## DISCUSSION

We demonstrated that HGI could significantly reduce D_2.0 cm3_ of the bladder and increase CTV_HR_D_90%_ and CTV_HR_V_100%_ in one IGABT plan without compromising target coverage or causing any associated complications. Moreover, an ADS of ≥2.60 was determined to be the optimum cut-off value to decide whether to perform HGI.

Brachytherapy is an important part of radiotherapy for cervical cancer [15], and a multicenter prospective cohort study of IGABT guided by MR imaging in locally advanced cervical cancer (EMBRACE-I) demonstrated that the median CTV_HR_D_90%_ was 90 (interquartile range: 85–94) GyEQD2, and the 5-year actuarial local control rate was 92% (95% CI: 90–93) at a median follow-up of 51 (interquartile range: 20–64) months [16]. Here, the incidence of grade 3 gastrointestinal, genitourinary and vaginal adverse events and fistulas was 18.4% (95% CI: 16.0–21.2), whereas that of grade 4 adverse events was 5.2% (95% CI: 4.0–6.9) according to CTCAE version 3.0; this indicated that serious brachytherapy-related morbidity from IGABT was low, despite the high dose delivered to the target. In contrast, IGABT using CT is more widespread than MR imaging in Japan [17]. Tumor delineation on CT images can potentially lead to an overestimation of tumor width [18]. Several Japanese investigations on IGABT utilizing CT scans have indicated that the CTV_HR_D_90%_ was lower compared to other studies. Despite this, the local control rate remained comparable or slightly lower. However, it’s noteworthy that the adverse event rate was lower than what was observed in the EMBRACE-I trial [19–21]. To combine the multiple advantages of these studies, which implies reducing adverse events while increasing CTV_HR_D_90%_ on IGABT using CT, HGI can be an effective method.

HGI can reduce bladder doses. Murakami *et al.* [3] investigated the usefulness of HGI in nine patients who underwent HGI in the RVS and VVS midway through a series of brachytherapy and found that D_2.0 cm3_ of the bladder per fraction was significantly lower in the group with HGI in the VVS than in that without HGI (449 [range, 416–566] cGy vs. 569 [range, 449–647] cGy, *P* = 0.033), with no compromising of target coverage. This finding is consistent with our study. However, the dose to the rectum seemed to decrease with HGI, but the difference was not statistically significant. This outcome could be attributed to the suboptimal injection position or failure to incorporate changes within the same individual, i.e. under the same conditions when analyzing dosimetric parameters. Iijima *et al.* performed the configuration analysis to determine the optimal location and volume of hyaluronate gel in the RVS or VVS for effective dose reduction to the OARs. They demonstrated that a gel spacer volume >10 cm^3^ is adequate to reduce the dosage for the OARs, provided that its craniocaudal length extends beyond the active length of the cylinder applicator and its gravity point is positioned at the midpoint between the OAR and cylinder applicator [5]. In the present study, 11 plans involved HGI utilizing cylinder applicators. However, in eight of these cases, the plans failed to meet the predetermined criteria for optimal injection positioning, which would have resulted in a dose reduction. Additionally, we analyzed and assessed the impact of HGI on individual patients. All participants underwent at least one plan with and without HGI (in total, 13 patients were included, accounting for a total of 50 plans). For each patient’s plan, we computed the mean value of each dosimetric parameter for plans both with and without HGI under identical conditions for that individual. Then, the Wilcoxon signed-rank test was performed to identify any significant difference in the dosimetric parameter between the plans with and without HGI, and the results indicated that HGI significantly increased CTV_HR_D_90%_. In contrast, it decreased the ADS and D_2.0 cm3_ in the rectum and bladder (Supplementary Table 1). However, the results of this statistical analysis were not definitive, given the small sample size and averaging of multiple measurements. Regarding the small intestine and sigmoid colon, hyaluronate gel injected into the RVS or VVS cannot reach the peritoneal cavity, where the majority of the sigmoid colon and small intestine are located, unless the needle punctures the peritoneum. In the absence of HGI in the peritoneal cavity and meso-sigmoid [22] or proximity between the sigmoid colon or small intestine and RVS or VVS, HGI is unlikely to contribute to the dose reduction of these organs. Nevertheless, the ADS formula accounts for the small intestine and sigmoid colon surrounding the uterus during the dose adjustment process.

This study demonstrated that adhering to the total dose constraint is possible even when the OAR doses are high or the CTV_HR_ doses are insufficient for the first IGABT without HGI. HGI is added after the second or subsequent IGABT sessions. This outcome may reduce the material costs associated with HGI, which medical providers are currently bearing. Suvenyl® is absorbed in 2–3 days [6] and should be injected at each IGABT session, and each HGI with Suvenyl® costs ~6000 Japanese yen for materials. Therefore, to help reduce the material costs associated with HGI, this study proposed the ADS and indicated that an ADS of ≥2.60 is an indicator to be considered for HGI implementation.

The ADS could have potential for improvement. In the IGABT plan, the ideal dose distribution is developed by evaluating the CTV_HR_D_90%_ and D_2.0 cm3_ of the OARs along with CTV_HR_V_100%_, representing the minimum dose covering 98% of the CTV_HR_, or the dose of the intermediate-risk CTV as defined by Haie-Meder *et al.* [23]. Tamaki *et al.* [24] demonstrated that the contributions of the EBRT dose after CS implementation to the CTV_HR_D_90%_, D_2.0 cm3_ of the bladder, and D_2.0 cm3_ of the rectum were 13–35%, 11–16% and 5–6% of the doses for a shielding width of 40 mm, respectively. However, the ADS formula proposed in this study incorporates only certain indices for quick and efficient decision-making regarding the adoption of HGI in clinical practice. Notably, the EBRT dose to the CTVHR, bladder and rectum post-CS was designated as 0 Gy within this formula.

**Fig. 4:**
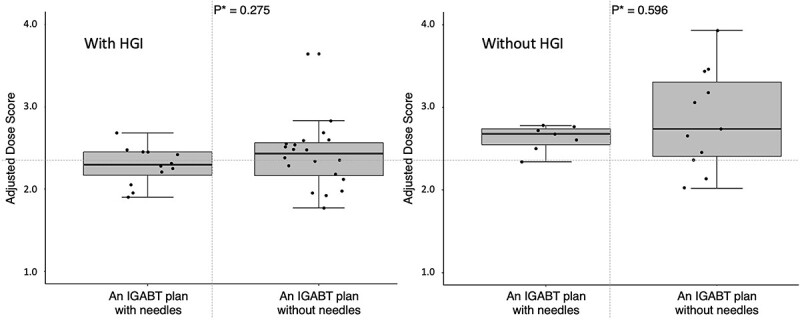
The box-whisker and jitter plots for the distribution of the ADS between the IGABT plans with and without HGI groups and with and without interstitial needles. ADS = adjusted dose sore, IGABT = image-guided adaptive brachytherapy, HGI = hyaluronate gel injection. ^*^The ADS of the two groups were compared using the Wilcoxon rank-sum test.

Furthermore, a significant concern is whether IC/IS brachytherapy and HGI are replicable at other institutions. Particularly for IC/IS brachytherapy, the effect of interstitial needle placement on the ADS may become more significant than the presence or absence of HGI. In this study, eight patients (31 IGABT plans) underwent IC/IS brachytherapy. Notably, no significant difference in the ADS was observed between groups utilizing interstitial needles or not, with or without HGI. Therefore, the needle position had minimal impact on the ADS (Fig. 4). However, this result is only true for institutions that are proficient in these techniques. Since acquiring proper needle placement and HGI techniques requires practice, it cannot be guaranteed that all institutions will offer comparable treatment; that is, the ADS may still be greatly influenced by needle insertion and HGI techniques when the same treatments are performed at multiple institutions. Initially, as previously noted, even with our technique, the positioning of the hyaluronate gel was not optimal in all IGABT plans involving HGI. Given these considerations, the ADS remains inadequate for widespread adoption in clinical practice. It is imperative to validate and generalize the ADS through a large-scale, multicenter prospective study encompassing institutions proficient in these techniques as well as those that are not.

This study has some limitations. First, the dosimetric superiority of HGI and the utility of the ADS were only demonstrated in this study, and it remains unclear whether this dosimetric superiority would translate into clinically significant advantages. Therefore, we plan to conduct follow-up studies over a long period to resolve these concerns. Second, the production of Suvenyl® was stopped at the end of 2023. As an alternative pelvic spacer to Suvenyl®, Muramoto *et al.* [25] reported the usefulness of MucoUp®, which has a lower molecular weight. We are also considering the use of another hyaluronate gel with a lower molecular weight than Suvenyl® for performing HGI (jRCTs071230118). Given its lower molecular weight and viscosity, adjusting the infusion volume of the alternative hyaluronate gel would be necessary to achieve a comparable effect to Suvenyl®. However, we believe that substituting Suvenyl® with a lower molecular weight hyaluronate gel may be feasible. Third, this retrospective study involved a limited number of patients. In addition, no significant difference was noted in the CTV_HR_ between plans with and without HGI, even when analyzed under the same patient conditions, but this could not be ruled out due to the small number of cases. This factor may have contributed to the ease of adhering to the dose constraints for the OARs and CTV_HR_ in the later IGABT sessions with HGI.

In conclusion, our findings suggest that HGI can effectively decrease D_2.0 cm3_ in the bladder while simultaneously increasing CTV_HR_D_90%_ and CTV_HR_V_100%_ in a single IGABT plan. Additionally, HGI can be introduced after a second or subsequent IGABT session, maintaining adherence to prescribed dose constraints. Furthermore, the ADS can be a useful indicator for the timing of HGI implementation.

## Supplementary Material

Supplementary_Table_1_rrae031

## Data Availability

The datasets used and/or analyzed during the current study are available from the corresponding author upon reasonable request.
