# Supplementary material for: Effective timing of hyaluronate gel injection in image-guided adaptive brachytherapy for uterine cervical cancer: a proposal of the ‘adjusted dose score’
Source: J Radiat Res. 2024 May 13;65(3):393–401. doi: 10.1093/jrr/rrae031 (PMC11115467; doi:10.1093/jrr/rrae031)
Supplement: Supplementary_Table_1_rrae031 [file supplementary_table_1_rrae031.docx]

| **Supplementary Table 1** Comparison of the dosimetric parameters, ADS, and CTV_HR_ for a single IGABT session between IGABT plans with and without HGI in the same individual | | | | |
| --- | --- | --- | --- | --- |
| **Parameters** | **IGABT plans without HGI**  **(number of patients=13)** | **IGABT plans with HGI**  **(number of patients=13)** | **Δ**  **(with HGI - without HGI)** | ***p***  **(Wilcoxon signed-rank test)** |
| CTV_HR_D_90%_ (GyEQD2) |  |  |  |  |
| Median (IQR) | 7.82 (7.41–8.46) | 8.54 (8.02–8.67) | 0.72 | 0.033* |
| CTV_HR_V_100%_ (%) |  |  |  |  |
| Median (IQR) | 100.0 (99.9–100.0) | 100.0 (100.0–100.0) | 0.00 | 0.588 |
| D_2.0 cm3_ of the rectum (GyEQD2) |  |  |  |  |
| Median (IQR) | 4.58 (3.15–4.93) | 3.47 (2.72–4.92) | -1.11 | 0.036* |
| D_2.0 cm3_ of the bladder (GyEQD2) |  |  |  |  |
| Median (IQR) | 5.81 (5.58–5.97) | 5.37 (4.36–5.83) | -0.44 | 0.040* |
| D_2.0 cm3_ of the sigmoid colon (GyEQD2) |  |  |  |  |
| Median (IQR) | 4.82 (4.16–5.13) | 4.78 (4.42–4.93) | -0.04 | 0.946 |
| D_2.0 cm3_ of the small intestine (GyEQD2) |  |  |  |  |
| Median (IQR) | 3.73 (2.50–4.38) | 3.29 (1.78–4.05) | -0.44 | 0.787 |
| ADS |  |  |  |  |
| Median (IQR) | 2.75 (2.45–3.18) | 2.28 (2.19–2.47) | -0.47 | 0.001* |
| CTV_HR_ (cm^3^) |  |  |  |  |
| Median (IQR) | 29.56 (17.42–54.13) | 22.52 (16.47–46.60) | -7.04 | 0.094 |
| The mean value of each dosimetric parameter for the plans with and without HGI for the same individual is calculated, and the corresponding data is compiled. ADS, adjusted dose score; CTV_HR_, high-risk clinical target volume; IGABT, image-guided adaptive brachytherapy; HGI, hyaluronate gel injection; CTV_HR_D_90%_, the minimum dose covering 90% of the CTV_HR_; EQD2, the equivalent dose in 2 Gy fractions; CTV_HR_V_100%_, the percentage of CTV_HR_ receiving 100% of the prescription dose; D_2.0 cm3_, the most exposed 2.0 cm^3^; IQR, interquartile range. *Statistically significant (*P* <0.05). | | | | |
